# A review of carbon mineralization mechanism during geological CO_2_ storage

**DOI:** 10.1016/j.heliyon.2023.e23135

**Published:** 2023-12-02

**Authors:** Kyuhyun Kim, Donghyun Kim, Yoonsu Na, Youngsoo Song, Jihoon Wang

**Affiliations:** Department of Earth Resources and Environmental Engineering, Hanyang University, Seoul, 04763, South Korea

**Keywords:** Carbon mineralization, CCS, Mineral trap, Basalt, Sandstone

## Abstract

The CO_2_ trap mechanisms during carbon capture and storage (CCS) are classified into structural, residual, solution, and mineral traps. The latter is considered as the most permanent and stable storage mechanism as the injected CO_2_ is stored in solid form by the carbon mineralization. In this study, the carbon mineralization process in geological CO_2_ storage in basalt, sandstone, carbonate, and shale are reviewed. In addition, relevant studies related to the carbon mineralization mechanisms, and suggestions for future research directions are proposed. The carbon mineralization is defined as the conversion of CO_2_ into stable carbon minerals by reacting with divalent cations such as Ca^2+^, Mg^2+^, or Fe^2+^. The process is mainly affected by rock types, temperature, fluid composition, injected CO_2_ phase, competing reaction, and nucleation. Rock properties such as permeability, porosity, and rock strength can be altered by the carbon mineralization. Since changes of the properties are directly related to injectivity, storage capacity, and stability during the geological CO_2_ storage, the carbon mineralization mechanism should be considered for an optimal CCS design.

## 1 Introduction

The CO_2_ concentration in the atmosphere has increased at an unprecedented rate, primarily owing to human activities. According to Friedlingstein (2022), the atmospheric CO_2_ concentration, which was approximately 280 ppm in 1750, has reached 414.72 ppm in 2021 and has increased at a rate of 2.4 ppm per year in the 2010s [1]. In an attempt to reduce CO_2_ emissions by more than 45 % compared to 2010 by 2030 to mitigate climate change and to limit the global average temperature rise within 1.5 °C by 2100, as recommended by Intergovernmental Panel on Climate Change (IPCC), the carbon capture and storage (CCS) technology is considered as the most viable method to permanently sequestrate massive amount of CO_2_ [2]. The International Energy Agency (IEA) has suggested that CCS will be responsible for 18 % of global CO_2_ reductions by 2070 in the sustainable development scenario [3]. In addition, many countries, including the United States, United Kingdom, Germany, Japan, and South Korea, have chosen CCS technology as a key strategy to meet the nationally determined contribution to reduction in its CO_2_ emissions [4].

According to the Global CCS Institute, the total injection capacity of commercial CCS projects extends to 243.97 Mtpa, a 44 % increase compared to that in 2021 [5]. CCS project can be classified according to its CO_2_ source, storage type, and annual injection volume. While CO_2_ sources in the past were primarily gas-processing facilities, recent trends have shown that CO_2_ sources have diversified into ethanol production plants, hydrogen production plants, and power plants. Because the CO_2_ captured by each industry has a different chemical composition, proper transport and geological storage of CO_2_ according to its source is necessary [3]. Geological storage types are mainly classified into Enhanced Oil Recovery (EOR)/Enhanced Gas Recovery (EGR) sites, depleted reservoirs, saline aquifers, and unmineable coal seams [6,7]. To select an appropriate storage target, geothermal, geohazard, hydrodynamic, basin maturity, and economic, societal and environmental factors should be taken into account [7]. Today, deep saline formations are the most common type of CO_2_ storage reservoir at all stages, from development to completion [8]. Despite this trend, depleted reservoirs and EOR sites are still significant for future project development, particularly in regions with developed oil and gas industries, such as the North Sea, the United States, and the Middle East. The average injection rate of ongoing projects was slightly above 1 Mtpa in 2022 [5]. However, many CCS projects in the development stage have injection rates of approximately 5 Mtpa, which is expected to dramatically increase the annual storage capacity of CO_2_ through CCS [8]. Although the most common storage targets are deep saline aquifer and depleted hydrocarbon reservoir aiming at structural trap as the primary trapping mechanism, recent studies and pilot projects have demonstrated the feasibility of storing CO_2_ in basalt formations using mineral traps.

**Fig. 1:**
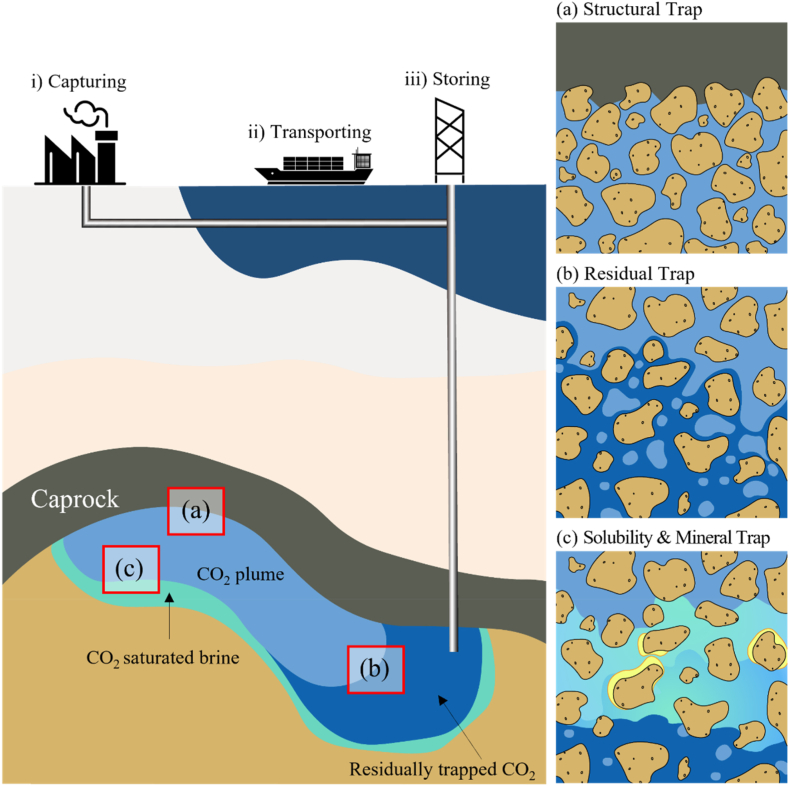
CCS procedure and CO_2_ trap mechanism. (a) Structural trap, (b) Residual trap, (c) Solubility & Mineral trap.

**Fig. 2:**
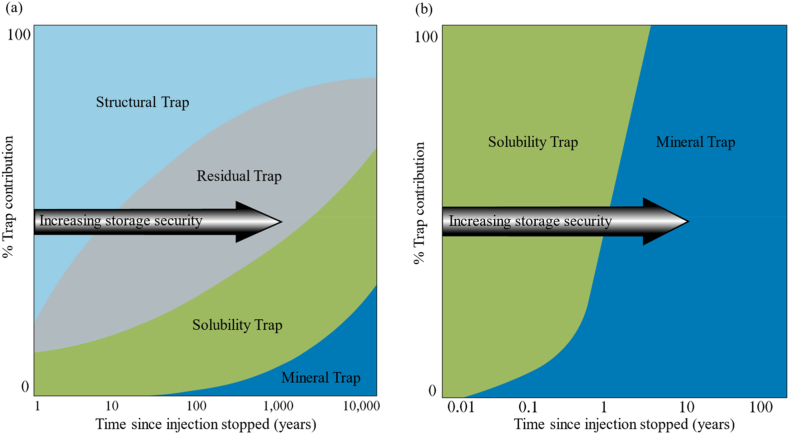
Trap contribution of CO_2_ for each trap mechanism with time in (a) a sedimentary basin (modified after IPCC [11]) and (b) in a basalt formation (modified after Raza and Glatz [11,12,16]).

The trap mechanisms are categorized into structural, residual, solubility, and mineral traps (Fig. 1(a, b, c)). By the structural trap mechanism, CO_2_ is physically trapped in a permeable and porous rock formation confined by impermeable boundaries such as cap rocks and seals. When CO_2_ is trapped in pores or rock fractures by the capillary pressure, it is defined as the residual trap. The solubility trap described as the CO_2_ dissolved in the formation fluids and trapped in the formation. The mineral trap mechanism is defined as the dissolved CO_2_ reacts with metal ions in subsurface rocks to form stable carbonate minerals [9]. The mineral trap mechanism is known to be the most stable trap mechanism because it may form stable carbonate minerals by the carbon mineralization [10]. However, it is not normally considered as a dominating one as it takes a very long time in a sandstone layer, the most common rock type of targeted geological structures such as deep saline aquifers and depleted hydrocarbon reservoirs [9,11]. This is because the carbon mineralization is the reaction between the CO_2_ and the divalent metal ions including Ca^2+^, Mg^2+^, or Fe^2+^, which sandstone generally has a very low content. Fig. 2(a) shows the contributions of each trap mechanism at time since injection stops in a sedimentary basin. The structural trap has the largest contribution to the CO_2_ storage initially, and the contribution of the other trap mechanisms increases with time as well as the storage security. The trap mechanism that occurs in the latest time period is the mineral trap, which is sometimes expected to begin the contribution after thousands of years after the injection finishes [12]. Alternatively, CO_2_ can be stored in a reservoir composed of reactive rocks such as basalt, which has a rapid mineral trap rate. According to the CarbFix project, which are representative CCS pilot project aiming at basalt formations, CO_2_ dissolved in water was injected. Once dissolved in water, CO_2_ is no longer buoyant does not migrate to the surface. Therefore, the structural and residual traps are not considered as shown in Fig. 2(b) [[13], [14], [15]]. It was found that the solubility trap was dominating in the early time, and the mineral trap became a dominating trap mechanism within two years [12]. Since the mineral trap mechanism is induced by the carbon mineralization, it needs to be taken into account when a geological CO_2_ storage is designed.

The carbon mineralization is generally divided into in-situ and ex situ mineralization in terms of its location. The in-situ carbon mineralization is performed by injecting CO_2_ into a highly reactive rock formation with high divalent cation content, such as a basalt. In addition, studies suggest that volcanogenic sandstones are preferable candidates for the in-situ mineralization as they contain a high percentage of volcanic rock fragments (VRF), which contain the highly reactive minerals. Zhang et al. (2013) showed that 80 % of the CO_2_ was mineralized when injected to a volcanogenic sandstone containing 10–20 % of the reactive minerals.

On the other hand, the ex-situ carbon mineralization occurs on the surface by the reaction of feedstock, such as fly ash. It is considered as a permanent method and no need to monitor [17,18]. The ex-situ carbon mineralization is divided into the direct and indirect methods. The representative direct methods are the gas-solid carbonation and direct aqueous mineral carbonation [19]. During the indirect mineral carbonation, Ca^2+^ or Mg^2+^ ions extracted from the reactive components react with the CO_2_ to form a desired carbonate [20]. The ex-situ carbon mineralization has been adopted globally, such as the Kimberlite mine in South Africa, the Mount Keith mine, and an open-pit nickel mine in Western Australia [[21], [22], [23]]. According to Kelemen [2], the in-situ carbon mineralization is considered as a more economical process because additional process for the reactant transport is not required if an appropriate formation is targeted.

The in-situ carbon mineralization has been dealt with many researchers and most of the studies have solely focused on basalt formations due to its high content of metallic cation [16,24,25]. However, the carbon mineralization in different rock types including sandstone, carbonate and shale should be considered as the various types of CO_2_ storage are recently targeted, such as CO_2_ injection in saline aquifers and depleted hydrocarbon reservoirs, and CO_2_ injection to enhance hydrocarbon recovery. Moreover, even if the target formation does not contain high content of the reactive minerals, the long-term carbon mineralization mechanism needs to be investigated for a more accurate monitoring and predicting the distribution of the injected CO_2_.

In this study, relevant studies focusing on the in-situ carbon mineralization during geological CO_2_ storage in basalt, sandstone, carbonate, and shale are reviewed. In tion 2, the detailed carbon mineralization mechanism is addressed. tion 3, 4 and 5 discuss relevant studies focusing on the in-situ carbon mineralization mechanisms in basalt, sandstone, carbonate, and shale formations.

## 2 Carbon mineralization mechanism

**Fig. 3:**
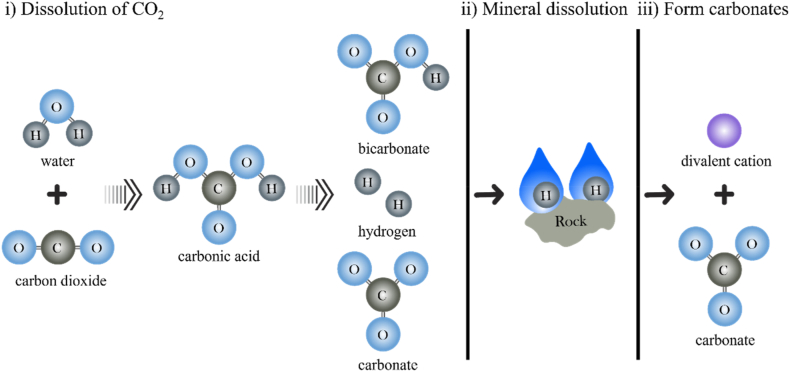
Carbon mineralization process (modified after Sandalow [32]).

The carbon mineralization refers to the CO_2_ reacted with divalent cations such as Ca^2+^, Mg^2+^ or Fe^2+^ to form stable carbonate minerals [26]. The process is divided into three steps (Fig. 3): i) dissolution of gaseous CO_2_ in aqueous solution to form bicarbonate and carbonate ions, ii) mineral dissolution producing such as Ca^2+^, Mg^2+^, and Fe^2+^-rich solutions, and iii) formation of stable calcite (CaCO_3_), magnesite (MgCO_3_), or siderite (FeCO_3_) through ionic reactions at high pH condition [27]. The detailed reaction steps are listed in Table 1. After injected, CO_2_ dissolves in the formation water (Equations (1) and (2)). As the CO_2_ dissolves in the formation water, the pH decreases up to typically 3.0–5.0 (Equations (3) and (4)). The low pH environment promotes the dissolution of divalent cation-bearing minerals [25]. As CO_3_^2−^ diffuses and is diluted in the formation water, the pH increases. Finally, carbonate ions and divalent cations form carbonate minerals such as calcite, magnesite, or siderite (Equations (5), (6), and (7)) [28]. In addition, the process may also produce other various carbonate minerals, such as aragonite (CaCO_3_), ankerite (Ca(Fe, Mg, Mn) (CO_3_)_2_), dolomite (CaMg(CO_3_)_2_) [[29], [30], [31]].

**Table 1:** Carbon mineralization steps.

i) Dissolution of CO_2_ to release hydrogen (H^+^) and carbonate (CO_3_^2−^) ions in solution	
$CO_{2}\left(g\right)⇋CO_{2}\left(aq\right)$	(1)
$CO_{2}\left(aq\right)+H_{2}O⇋H_{2}CO_{3}\left(aq\right)$	(2)
$H_{2}CO_{3}\left(aq\right)⇋HCO_{3}^{-}\left(aq\right)+H^{+}\left(aq\right)$	(3)
$HCO_{3}^{-}\left(aq\right)⇋CO_{3}^{2-}\left(aq\right)+H^{+}\left(aq\right)$	(4)
ii) Mineral dissolution to release Ca^2+^, Mg^2+^, Fe^2+^ cations into solution	
iii) Ionic reactions to form stable carbonates at high pH condition	
$CO_{3}^{2-}\left(aq\right)+{Ca}^{2+}\;⇋CaCO_{3}\left(s\right)$	(5)
$CO_{3}^{2-}\left(aq\right)+{Mg}^{2+}\;⇋MgCO_{3}\left(s\right)$	(6)
$CO_{3}^{2-}\left(aq\right)+{Fe}^{2+}\;⇋FeCO_{3}\left(s\right)$	(7)

The rock type and temperature condition have an important role in the in-situ carbon mineralization process. Mafic or ultramafic rocks are appropriate candidates for the process due to its high concentrations of the divalent cations required for calcite, dolomite, and magnesite formation. Zhang and DePaolo suggested that the rate of the carbon mineralization depends not only on the abundance of the divalent cations but also the rate at which the cations are released from minerals [33]. According to Saldi [34], dissolved Ca^2+^ readily precipitates in aqueous fluid at temperature below 280 °C. Meanwhile, dissolved Mg^2+^ precipitates as carbonate magnesite and dolomite at temperatures above 80 °C. However, the temperatures at which dissolved carbonate precipitates by reacting with Fe^2+^ remains unclear [25,34].

Other factors that affect the carbon mineralization include the CO_2_ phase during injection, partial pressure of CO_2_ (pCO2), and silicate precipitation rate. Pearce et al. (2021) [35] claimed that the injection of CO_2_ dissolved-brine, which provides cations, may be favorable for accelerating carbon mineralization compared to injecting CO_2_ in the gaseous state. For example, in the CarbFix project, CO_2_ was dissolved in groundwater before or during its injection, and carbon mineralization occurred within two years [15]. Fe-rich carbonates mainly precipitate at a high pCO2 whereas Ca-rich carbonates mainly form at a low pCO2. These characteristics affect the mineral distribution of the precipitated carbonate during the core flooding experiments. In general, the precipitation of Fe-carbonate mainly occurs at the center of the CO_2_ plume, whereas the precipitation of Ca-carbonate actively occurs outside, at lower pCO_2_ and higher pH [30]. Similarly, Voigt et al. [29] found that magnesite was actively formed under higher pCO_2_ (16 bar), whereas calcite was actively formed under lower pCO_2_ conditions (2.5 bar). The released divalent cations are consumed for the secondary precipitation of silicate minerals (zeolite and chlorite) and clay minerals (smectite, kaolinite), and carbonate minerals [36]. Secondary silicate precipitates usually occur in a wider area than carbonate minerals, and can deteriorate injectability by clogging pores [37].

## 3 The carbon mineralization in basalt

**Fig. 4:**
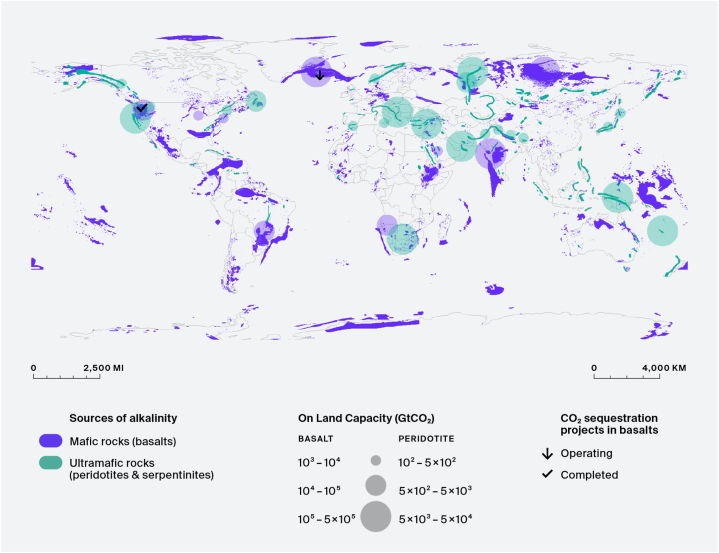
Location of mafic and ultramafic rocks for carbon mineralization with the CO_2_ sequestration potential [39].

CCS projects in basalt formations aim at the solubility and mineral traps as the dominating trap mechanisms in a short period. Since the high carbon mineralization rate in basalt improves the storage security, basalt formations have recently attracted attention as a host rock for CCS projects in spite of its highly heterogeneous distributions of permeability and porosity [7,16]. Another advantage of CCS in basalt formations are that its wide distribution in the Earth's crust. According to Aminu et al. [7] that basaltic rocks form approximately 8 % of the continents and much of the ocean floor. Fig. 4 indicates basalt formations adjacent to major CO_2_ point emission sources [38,39]. In particular, CCS in basaltic oceanic crust is worth considering because saline water required for CO_2_ injection can be easily secured [16]. The CarbFix and Wallula projects are renowned basalt CCS pilot projects that have identified CO_2_-basalt interactions and have performed continuous isotope monitoring. Although the projects are not yet commercial-scale, they show potential for large-scale CO_2_ storage in basalt formations [40].

Biotite (K(Mg, Fe)_3_(AlSi_3_O_10_) (F, OH)_2_), amphibole ((Ca, Mg, Fe)_7_Si_8_O_22_(OH)), pyroxene ((Ca, Mg, Fe)_2_Si_2_O_6_), and olivine ((Mg, Fe)_2_SiO_4_) consist of abundant Mg^2+^ and Fe^2+^ ions, which are easily found in mafic rocks such as basalt and gabbro. Plagioclase (CaAl_2_Si_2_O_8_) is another major mineral component of mafic rocks that contains abundant Ca^2+^ [16]. In addition to mineral components, basalt has amorphous materials called basaltic glass, which also contains abundant divalent cations including Ca^2+^, Mg^2+^, and Fe^2+^ [41]. The components are potential reactants for the carbon mineralization and provide the divalent cations required for carbonate precipitation under specific conditions [42,43]. The chemical equations of major dissolution mechanisms are presented as below. Forsterite (Mg_2_SiO_4_) is a magnesium endmember in the olivine solid solution (Equation (8)). Diopside (MgCaSi_2_O_6_) is a pyroxene mineral with a monoclinic crystalline structure (Equation (9)). Calcium plagioclase is a calcium endmember belonging to the plagioclase solid solution (Equation (10)).

(8) $${Mg}_{2}{SiO}_{4}+{4H}^{+}\to \;{2Mg}^{2+}+{2H}_{2}O+{SiO}_{2}\;\left(aq\right)$$

(9) $${MgCaSi}_{2}O_{6}+{4H}^{+}\;\to {Mg}^{2+}+{Ca}^{2+}+{2H}_{2}O+{2SiO}_{2}\;\left(aq\right)$$

(10) $${CaAl}_{2}{Si}_{2}O_{8}+{8H}^{+}\to \;{Ca}^{2+}+{2Al}^{3+}+{2SiO}_{2}\;\left(aq\right)+{4H}_{2}O$$

### 3.1 Experimental studies

Various experimental studies have been carried out to understand detailed mechanisms and behaviors of the carbon mineralization in basalt (Table 2). Injectability, storage capacity, and stability are major factors to consider in conventional CCS. For CCS in basalt formations, the reactivity and types of reactants and products are also important factors to consider, because the main mechanism of CCS in basalt is mineral trapping through carbon mineralization.

**Table 2:** Experimental studies of the carbon mineralization in basalt.

Author	Method	Lithology	Aqueous Matrix	Temperature (°C)	Pressure (MPa)	CO_2_ Phase	Time	Carbonate Precipitation	Remark
Giammar et al. (2005)	Batch reactor	Forsterite (San Carlos, Arizona)	MgCl_2_/NaHCO_3_ solution	30–95	0.1 and 10	CO_2_(g)	29 days (692 h)	Magnesite	Magnesite precipitation through nucleation effect in forsterite
Schaef et al. (2010)	Batch reactor	Newark Basin Basalt, Columbia River Basalt, Karoo Basalt	Water	100	8.9	scCO_2_	377 days	Magnesite, Siderite	Carbon mineralization in 5 types of volcanic basalt
Schaef et al. (2011)	Batch reactor	Central Atlantic Magmatic Province Basalt, Columbia River Basalt	water	34–137	7.5–31	scCO_2_/CO_2_ (aq)	180 days	Calcite, Aragonite	Compare reactivition rate between CO_2_ saturated water and water saturated CO_2_
Gysi and Stefansson (2012)	Batch reactor	Basaltic glass (Stapafell Mountain in SW Iceland)	Water	75–250	1.1–2.4	CO_2_(g)	125 days	Ankerites	Competing reactions under the high temperature condition
Rosenbauer et al. (2012)	Batch reactor	Tholeitic basalts (Juan de Fuca Ridge/Mt. Lassen)	NaCl brine	50–200	30	scCO_2_	179 days (4300 h)	Magnesite	Carbon mineralization by Mg content
Shibuya et al. (2013)	Reaction cell	Synthetic basalt	NaCl brine (Hadean/Archean seawater)	250 and 350	50	CO_2_(aq)	90 days (3 months)	Calcite	Basalt–seawater–CO_2_ interaction in the modern oceanic crust environment
Roy et al. (2016)	Fabricated Triaxial Chamber	Deccan Basalt	Cold water during coring	–	0.25	CO_2_(g)	30, 50, 70, and 90 days	Calcite	Comparison of sequestration potential under the basalt type with same formation
Adeoye et al. (2017)	Flow-through reactor Batch reactor	Columbia River Basalt, Serpentinized basalt (Valmont Butte, Colorado)	Pure water, 1.2 mM of [NaHCO_3_] and 13.8 mM of [NaCl] brine	45 and 100	10	CO_2_(aq)	42 days	Magnesite, Calcite	Trend of dissolution and precipitation by transport limitation effect
Luhmann et al. (2017)	Autoclave	Basalt (Eastern Snake River Plain, Idaho)	NaCl brine	150	15	scCO_2_	0.51–32.81 days	Siderite	Carbon mineralization under high pressure and temperature condition
Kanakiya et al. (2017)	Routine core analysis	Basalt (Auckland volcanic field)	Water equilibrated with rock samples for 62 days	100	4.5–5.5	CO_2_(g)	140 days	Ankerites	Sequestration potential by basaltic galss content and porosity
Xiong et al. (2017)	Batch reactor	Columbia River Basalt, Serpentinized basalt (Valmont Butte, Colorado)	Pure water	100	10	scCO_2_	240 days (40 weeks)	Siderite	Carbon mineralization in the fractured balsalt
Wolff-Boenisch and Galeczka (2018)	Flow-through reactor	Basaltic glass, Crystalline basalt (Stapafell Mountain in SW Iceland)	synthetic seawater given by Millero (2003)	90	0.6	CO_2_(g)	1 day (25h)	Calcite, Magnesite, Ca/Mg-carbonates	Competing reactions under the post-injection phase
Menefee et al. (2018)	Flow-through reactor	Serpentinized basalt (Valmont Butte, Colorado)	6.3 mM and 640 mM [NaHCO_3_] solution	100–150	10	scCO_2_	10–12 days	Calcite, Aragonite	Carbonate localization and geochemical gradient
Clark et al. (2019)	Batch reactor	Basaltic glass (Stapafell Mountain in SW Iceland)	Water	50	8	scCO_2_	0.5 days (12h)	No	Competing reactions by pH condition change
Marieni et al. (2020)	Batch reactor	Basalts (Juan de Fuca/Mid-Atlantic Ridges), Gabbro (Cyprus)	IAPSO Seawater	40	0.25 and 1.6	CO_2_(g)	20 days	No	Low temperature dissolution potential of balsalt and gabbro
Al-Yaseri et al. (2021)	Contact Angle	Basalt (Bunbury, Western Australia)	NaCl brine	24.85 and 49.85	0.1–20	scCO_2_	No	–	Wettability change of basalt under the high pressure and temperature condition
Voigt et al. (2021)	Batch reactor	Basaltic glass (Stapafell Mountain in SW Iceland)	North Atlantic Seawater	130	0.25	CO_2_(g)	210 days (7 months)	Calcite, Aragonite, Magnesite	Long-term reaction of basalt–seawater–CO_2_ interaction

Types of reactants and products, and the reaction rate depend on temperature and pressure condition**.** Xiong et al. [44] conducted batch experiments and found that siderite was formed at 100 °C whereas Ca–Mg–Fe carbonates were formed at 150 °C by a set of experiments in fractured basalt specimens. Rosenbauer et al. [45] discovered magnesite precipitation occurs most actively at 100 °C by comparative analyses with experimental and computational approach. They found that the reaction rate of Mg^2+^ was proportional to its concentration in basalt. Voigt et al. [29] conducted batch experiments to determine the carbon mineralization behaviors at 2 pCO_2_ conditions and found that calcite and aragonite are predominantly formed at 0.25 MPa pCO_2_, whereas magnesite is predominantly formed at 1.6 MPa pCO_2_. The results show that the temperature and pressure conditions significantly affect the rate of each carbon mineralization reaction and determine the types of precipitates. In addition, the CO_2_-water-basalt interaction becomes more active at higher temperatures and pressures.

There are studies focusing on effects of the rock type of the basalt formation on the CO_2_-water-rock interaction. Basalt generally consists of olivine, pyroxene, plagioclase, and basaltic glass. However, various types of basalts have undergone mineral alterations, which cause changes in the composition and grain size. Considering this, several studies have compared carbon mineralization according to the basalt type. Adeoye et al. [46] analyzed the types of carbon mineralization in the fractures of unaltered and serpentinized basalt specimens, and suggested that the size of the rock grains affects the rate of dissolution and precipitation. Discrete siderite crystals (nodules) were entirely formed in the fine-grained unaltered basalt (50–100 μm), whereas millimeter-scale siderite clusters were formed in the coarser-grained serpentinized basalt (100–150 μm). Marieni et al. [47] performed experiments to compare the reactivities of crystalline submarine basalts and highly altered gabbro. It was confirmed that only Ca^2+^ ions are originated from plagioclase in basalt, whereas Ca^2+^ ions from amphibole and plagioclase in gabbro. Kanakiya et al. [31] observed effects of the carbon mineralization on microstructure. They used three types of basalt specimens, which are originated from same formation, Auckland Volcanic Field, with different rock properties and mineral compositions. The results indicate that higher initial porosity and basaltic glass contents enhance dissolution and precipitation rates.

The fluid composition is also a key factor affecting the results of carbon mineralization behavior. Especially for CCS in basalt formations, studies are being conducted to inject CO_2_ dissolved water aqueous solutions to reduce the potential risk of leakage and increase reaction rate. Therefore, compositions of the injection fluid and formation water should be considered. Some of studies have revealed the advantages of using seawater as an injection fluid in terms of reaction rate and economic efficiency. Voigt et al. [29] observed a high reaction rate in a carbon mineralization experiment using North Atlantic Seawater. Wolff-Boenisch and Galeczka [36] performed reactivity test and revealed that continuous carbon mineralization can occur in the post-injection phase by using synthetic seawater with alkaline pH 8.1 as injection fluid. Based on experiments using weakly acidic fluid which mimics Hadean/Archean seawater (pH range 4.9–6.5), Shibuya et al. [48] observed decrease in divalent cations concentration and increase in calcite precipitation as the reaction progresses. In addition, there are studies focusing on compositions of formation water and phase of injected CO_2_. In a set of experiments using forsterite, Giammar et al. [49] found that magnesite precipitation occurred only when the brine contained MgCl_2_ and NaHCO_3_. Similarly, Menefee et al. [43] analyzed carbonate precipitation when the concentration of NaHCO_3_ in the brine was abnormally high (640 mM), and showed that NaHCO_3_ increases the CO_3_^2−^ concentration, pH, and consequently, the amount of carbonate precipitates. Clark et al. [30] performed a mineralization experiment using a column reactor and found that it is important to maintain the pH low (<6) during the initial injection phase to improve the mineralization reactivity. Schaef et al. [50] compared the morphology of carbonate precipitates formed by supercritical CO_2_ (scCO_2_) under high temperature and pressure conditions. They confirmed that the dissolved CO2 coated the surface of basalt when water saturated scCO_2_ is injected, while discrete nodules were created when CO_2_ saturated water is injected. Schaef et al. [51] conducted mineralization experiments on basalt formations using CO_2_ saturated water and CO_2_–H_2_S mixture saturated water. When the CO_2_ saturated water was injected, the Newark Basin basalt and the Karoo basalt respectively showed the highest and lowest reactivity. However, when the CO_2_–H_2_S mixture saturated water was injected, the carbon mineralization did not occur in the Newark Basin basalt and relatively high reactivity was observed in the Karoo basalt. These results indicate that the carbon mineralization reactivity strongly depends on the composition of the injection fluid, even in the same formation.

Several studies have suggested that other important factors must be considered. The precipitation of silicate minerals is a competing reaction for carbon mineralization by limiting pore space and divalent cations. The reactivities depend on the conditions of the target formations [30]. Gysi and Stefánsson [52] found that carbonate minerals other than calcite were not produced at temperatures above 150 °C. Instead, experimental results show that there is active precipitation of silicate minerals such as smectite and chlorite. Menefee et al. [43] observed that Fe-rich oxide coatings formed during Ca-rich carbonate precipitation. The result indicates that in a specific temperature and pressure range (100–150 °C and 10 MPa pCO_2_), Fe^2+^ oxidation is favored over carbonate mineralization. Voigt et al. [29] revealed that smectite precipitation occurred under low-pressure conditions (0.25 MPa), and it may limit well injectivity over time by pore clogging. Clark et al. [30] confirmed that Fe–Mg–Ca-carbonate minerals, Saponite and zeolite competitively precipitated in a large column reactor experiment. Wolff-Boenisch and Galeczka [36] performed flow-through reactor experiments at 90 °C and observed the precipitation of smectite, zeolite, and chlorite. The authors expected that the silicate precipitation would be more likely to occur as temperature of the most storage formations is lower than 90 °C. Therefore, silicate precipitation should be focused for field-scale geological CO_2_ storage target formation.

There are studies proposing the effect of nucleation, which mainly affects the reaction rate and distribution of carbonate precipitates. Giammar et al. [49] found that supersaturation conditions with respect to specific divalent cations does not guarantee carbonate precipitation. They revealed that initial nucleation must be considered which limit the rate of the magnesite precipitation. As a result, it only occurs under the critical degree of supersaturation (Saturation Index: 0.25–1.14 under 95 °C and 10 MPa pCO_2_). In subsequent experiments, they analyzed ion concentrations of effluent and observed that nucleation was accelerated in basalt-containing magnesite seeds. The results indicate that the mineral trap occur rapidly in basalts containing preexisting carbonate minerals. Menefee et al. [43] found that the secondary precipitation mainly occurs where the primary minerals exist as those provide divalent cations.

The pore structure also affects the distribution of carbonate precipitation by mass transport limitations. The advective flow has a short retention time and primarily occurs near the wellbore and in pore spaces with high conductivity. By contrast, diffusive mass transport has a long retention time and occurs in dead-end fractures. The diffusive transport causes a decrease in CO_2_ concentration and an increase in pH by the slow ion exchange. Therefore, areas with low conductivity have favorable conditions for the accumulation of carbonate precipitates [46]. Xiong et al. [44] conducted a set of experiments using fractured basalt specimens and found that the mass transport limitation and the sufficient surface area provide favorable conditions for accumulation of the carbonate precipitates (volume fraction change: 14.75 % at 150 °C). Menefee et al. [43] confirmed that the precipitation of clay minerals was dominant in advection dominating flow paths, while the precipitation of calcite and aragonite was dominant in dead-end fractures. The behaviors were caused by the difference in the pH of the locations where the reactions occur. Adeoye et al. [46] analyzed the differences between the advection flow and diffusive mass transport conditions using the flow-through and static batch experiments, respectively. It was found that dissolution dominated in the former condition, while the siderite precipitation dominated in the latter condition. In addition, using a batch reactor, Marieni et al. [47] observed CO_2_-water-rock interaction only occurred at the top-most layer of the rock powder and concluded that the effect of mass transport limitations should be considered for a more reliable outcome. Luhmann et al. [53] conducted flow-through experiments at 2 flow rate conditions and determined effects of the retention time on rock permeability change. It was found that the permeability increased at a higher flow rate (0.13 md to 0.22 md at 0.1 ml/min) and decreased at a lower flow rate (0.15 md to 0.12 md at 0.01 ml/min).

In terms of the stability of CCS in basalt formation, Guha Roy et al. [54] measured changes in the mechanical properties due to carbon mineralization using three types of Deccan basalt. The results confirmed that while reactivity was highest in Giant Plagioclase Basalt, the deterioration of integrity was also the most severe (90 % reduction in the compressive strength and 87.5 % reduction in the tensile strength). Al-Yaseri et al. [55] conducted a water-CO_2_ wettability experiment on the Western Australian basalt. It was found that the basalt formation was water-wet with the contact angle of 60° at 5 MPa and became CO_2_-wet at 15 MPa. The results imply that performance of the capillary and residual traps may deteriorate due to the improved CO_2_ mobility under high pressure conditions.

### 3.2 Numerical studies

There are studies computationally analyzing the carbon mineralization in basalt as shown in Table 3. Van Pham et al. [38] built a model using geochemical code, PHREEQC-2 and compared carbonate precipitation behaviors by temperature (40–100 °C). At the temperature of 40 °C, carbonate precipitation was limited to siderite, while Ca^2+^ were consumed for zeolite and oxide precipitation. On the other hand, at the temperature of 60–100 °C, magnesite and ankerite formation was observed. According to Jayne et al. [56], CCS aiming at basalt formations is very challenging to computationally model due to its heterogeneity and fracture-controlled hydraulic properties. The authors adopted the geostatistical reservoir characterization method with TOUGH3, which quantifies the permeability uncertainty and potential for the joint initiation and shear failure. It indicates that pore pressure can change up to 2200 m away from the injection well by CO_2_ injection during the first year while CO_2_ only migrates up to 400 m. Using a CrunchTope, a multicomponent reactive transport code, Menefee et al. [37] found that the advection flow promoted the carbon mineralization within 2 years since the injection started. However, the results show that the carbonate precipitates quickly redissolved as the primary minerals were depleted during the injection phase. In addition, it was found that the diffusion flow is a more favorable condition for the accumulation of carbonate precipitates after 2 years of the injection. The authors also analyzed the nucleation locations for each mineral, and insisted that calcite preferentially precipitated on pyroxene, magnesite was more uniformly distributed, and siderite preferentially precipitated on olivine. Erol et al. [57] constructed a 3-D reactive transport model using TOUGHREACT for 3 types of metamorphic formations, i.e., schist, schist-marble, and marble. Despite the precipitation of ankerite, the results showed that the carbon mineralization was limited because of the high initial mass fraction of CO_3_^2−^ in the schist and marble formations.

**Table 3:** Numerical studies of the carbon mineralization in basalt.

Author	Method	Lithology	Aqueous Matrix	Temperature (°C)	Pressure (MPa)	CO_2_ Phase	Time	Carbonate Precipitation	Remark
Van Pham et al. (2012)	Numerical modelling	Columbia River Basalt	Pure Water	40	10	scCO_2_	181 days	Magnesite	Sequestration potential by pressure and temperature changes
McGrail et al. (2017)	Field Results	Columbia River Basalt	Formation water	36	7.7	scCO_2_	2 years	Calcite	Rock property changes in Walula CCS project under the post-injection phase
Menefee et al. (2017)	Numerical modelling	Serpentinized basalt (Valmont Butte, Colorado)-	Pure Water	100	10	CO_2_ (aq)	42 days (6 weeks)	Calcite, magnesite, manganese**,** siderite	Effect of transport limitation and mineral spatial distribution
Jayne et al. (2019)	Stochastic approach	Columbia River Basalt	Pure Water	0–110	≤60	scCO_2_	1 year	–	Prediction of hydraulic properties considering heterogeity of basalt
Erol et al. (2022)	Numerical modelling	Schist/Marble	Geothermal fluid (pH: 6.14)	220	12.8 at 1600 m	scCO_2_	10 years	Ankerite	Sequestration potential in schist and marble

### 3.3 Field case

The CarbFix and Wallula projects are CCS pilot projects in basalt formations which demonstrated the possibility of the stable and permanent storage of CO_2_ in subsurface geological structures by the carbon mineralization. The CarbFix 1 project was conducted in the southwestern region of Iceland during 2012. In this project, 175 tons of pure CO_2_ captured from the Hellisheidi geothermal plant were dissolved into groundwater and injected into a basalt formation at 500 m. In the following stage, 73 tons of CO_2_–H_2_S gas mixture (75 % CO_2_–25 % H_2_S) were injected at the same layer [58,59]. The temperature and pressure at the target reservoir for both stages were approximately 20–50 °C and above 4 MPa [25]. The result indicates over 95 % of the CO_2_ was mineralized and converted into carbonate minerals in less than 2 years. The CarbFix 2 was upscaled from the original CarbFix 1 project with same CO_2_ source for a field-scale analysis. From 2014 to 2017, 23,200 tons of CO_2_ was injected to southern Hengill volcanic system, which consist of fractured and hydrothermally altered basalts. The depth and temperature of the injected layer were increased to 750 m and over 250 °C, respectively. Consequently, it was found that over 60 % of CO_2_ was stored by the mineral trap mechanism during the injection period, implying that the basalt formation is a promising candidate for the large-scale CCS project [60].

The Wallula project is another pilot project, which injected 977 tons of scCO_2_ into the Columbia River Basalts in Wallula, Washington, over a 3-week period in 2013. The initial temperature and pressure of the formation were 36 °C and 7.7 MPa, respectively. It was identified about 60 % of injected CO_2_ was sequestered by the mineral trap within 2 years [40,61].

## 4 The carbon mineralization in sandstone

Sandstone is a common host rock for hydrocarbon reservoirs and a favorable target for CCS due to its high porosity and permeability. Commercial CCS projects, such as Sleipner and Snøhvit, targeted sandstone reservoirs. In addition, the Illinois Industrial CCS projects captured CO_2_ from Archer Daniels Midland's corn ethanol plant and stored 1 Mtpa of CO_2_ into the Mount Simon sandstone layer [6]. In South Korea, a pilot test of CO_2_ injection was conducted in the Pohang Basin to study CO_2_ sequestration in saline aquifers [4,62,63].

As CO_2_ is dissolves in the formation water it becomes a weak acid, and it reacts with the minerals in the reservoir to form bicarbonate ions [11]. Mineralogical alterations mainly appear in the silicate and carbonate-cemented grain-to-grain contacts in the pore space [64]. The reactions for each mineral are as follows,

For Ca-rich feldspar, anorthite (CaAl_2_Si_2_O_8_) dissolves in acid environments, emitting ions [65]:

(11) $$Ca{Al}_{2}{Si}_{2}O_{8}+8H^{+}↔{Ca}^{2+}+2{Al}^{3+}+2H_{4}SiO_{4}$$

The precipitation of anorthite produces calcite and kaolinite, trapping CO_2_ in the mineral phase [66,67]:

(12) $$Ca{Al}_{2}{Si}_{2}O_{8}+{CO}_{2}+2H_{2}O↔{CaCO}_{3}+{Al}_{2}{Si}_{2}O_{5}{\left(OH\right)}_{4}$$

For K-rich feldspar, the dissolution of dolomite and K-feldspar buffers the pH, and the latter initiates kaolinite precipitation [65]:

(13) $$KAl{Si}_{3}O_{8}+4H^{+}+4H_{2}O↔K^{+}+{Al}^{3+}+3H_{4}SiO_{4}$$

(14) $$2KAl{Si}_{3}O_{8}+2H^{+}+9H_{2}O↔{Al}_{2}{Si}_{2}O_{5}{\left(OH\right)}_{4}+2K^{+}+4H_{4}SiO_{4}$$

For K-rich feldspar in saline solutions, a different mineral, dawsonite (NaAlCO_3_(OH)_4_), is produced during the precipitation process [66,68]:

(15) $$KAl{Si}_{3}O_{8}+{Na}^{+}+{CO}_{2}+H_{2}O↔NaAlCO_{3}{\left(OH\right)}_{2}+3SiO_{2}+K^{+}$$

K-feldspar also reacts with Mg-chlorite in a Mg-carbonate precipitation reaction [66]:

(16) $$KAl{Si}_{3}O_{8}+2.5{Mg}_{5}{Al}_{2}{Si}_{3}O_{10}{\left(OH\right)}_{8}+12.5CO_{2}↔{KAl}_{3}{Si}_{3}O_{10}{\left(OH\right)}_{2}+1.5{Al}_{2}{Si}_{2}O_{5}{\left(OH\right)}_{4}+12.5MgCO_{3}+4.5SiO_{2}+H_{2}O$$

Albite (NaAlSi_3_O_8_) reacts with the carbonic acid, as described in the following reaction [65]:

(17) $$NaAl{Si}_{3}O_{8}+{4H}^{+}+4H_{2}O↔{Na}^{+}+{Al}^{3+}+3H_{4}SiO_{4}$$

The trapping mechanism of albite forms Na-smectite, as described in Equation (18) [66,67]:

(18) $$7NaAl{Si}_{3}O_{8}+{6CO}_{2}+{6H}_{2}O↔Na-smectite+6HCO_{3}^{-}+10SiO_{2}+6{Na}^{+}$$

It is important to determine the effect of the carbon mineralization on property change of the sandstone. This is because the carbonic acid weakens the cementing strength of rock grains, causing particles to fall off the matrix, becomes suspended in pore spaces, and alters the pore skeleton [69,70]. The precipitation of minerals also alters the pore skeleton; therefore, defining the effect of mineralization may become a challenge.

The carbon mineralization behavior in sandstones strongly depends on the mineralogical composition. Quartz is the main mineral in many sandstones, and CO_2_ mainly reacts with feldspar, anorthite, albite, smectite described above. Audigane et al. [71] used TOUGHREACT to measure the geochemical reactivity of Utsira Sand, which contains 0.763 of the volume fractions of quartz. The authors concluded that the geochemical reactivity of the mineral trap had a porosity reduction of 0.01. Pearce et al. [69] used sandstone from the Surat Basin in Australia with 0.733 of the volume fractions of quartz and observed minor porosity changes were observed using micro-computed tomography (micro-CT). In addition, no major alterations in minerals were observed by using quantitative evaluation of materials by scanning electron microscopy (QEMSCAN) and scanning electron microscopy (SEM). The results concluded that quartz-rich sandstones were not affected by the carbon mineralization in 1537 h. Using TOUGHREACT, Zhang et al. [72] investigated the trade-off between higher reactivity and lower porosity and permeability. Volcanogenic sandstone, which has abundant divalent cation-bearing minerals were observed. The results show that up to 80 % mass of rock can be mineralized within 1000 years if the formation contains 10–20 % reactive minerals. In addition, it was found that 1 Mtpa of CO_2_ can be injected per well with the sufficient injectivity. Hangx et al. [73] concluded that less quartz-cemented sandstones may have lower strength than calcite-dissolution-induced sandstones, and that a short-term effect of the CO_2_ injection on the mechanical properties should be investigated.

Other factors also affect carbon mineralization. Yanzhong et al. [74] concluded that calcium chloride (CaCl_2_) in aquifers can be a source of the Ca^2+^ for the carbon mineralization in sandstones and that the total geologic CO_2_ storage capacity can be improved by adding NaCl, KCl and MgCl_2_ in the injection fluid. Al-Yaseri et al. [75] concluded that the clay quantity, salt type, salt concentration, acidity, distribution, and rock structure affect the permeability and porosity. Permeability changes of 11 % and 23 % was observed for two Berea sandstones (low clay content), but no permeability change in Bandera Gray sandstones (high clay content). Marbler et al. [76] conducted geochemical and geomechanical studies in 3 types of sandstones in from the North Germain Basin. Not only does the CO_2_-rock interaction affect the rock matrix and cements, but it also changes the mechanical properties, such as friction angle, cohesion, and uniaxial compressive strength. Co-injection of SO_2_ or H_2_S also affects the mineralization process, as these enhance pyrite (FeS_2_) precipitation [77].

### 4.1 Experimental studies

Experimental studies focusing on the carbon mineralization in sandstone are summarized in Table 4. Although significance of the carbon mineralization in sandstone is still debatable, there are various studies indicating where the effect is considerable or not. Marbler et al. (2013) [76] investigated the carbon mineralization behavior of sandstones from the North German Basin by exposing the specimens to scCO_2_ and brine for 2–4 weeks in an autoclave system. It was found that alterations occurred in both the carbonate and silicate cements of the sandstones. In addition, it was observed that secondary carbonate precipitation in the pore spaces and the reactions alter the pore space, resulting in changes in friction angle, cohesion, uniaxial compressive strength. For specimens treated with the scCO_2_-brine mixture, precipitation was observed by SEM and the rock strength was reduced. Tang et al. [65] injected N_2_-, CH_4_-, and CO_2_-saturated water through core flooding sandstone specimens extracted from the Dongfang (DF) gas fields in China. SEM, energy-dispersive spectrum (EDS), X-ray diffraction (XRD), and flame atomic absorption spectrometry (FAAS) was used to analyze the property alterations due to CO_2_-brine-rock interaction. It was concluded that the CO_2_-brine-rock interaction have a negative impact on CO_2_ injectivity and storage, as minerals can precipitate and accumulate in the pore throats to ultimately decrease the permeability. However, the effect is minor in high-porosity and high-permeability reservoirs because precipitated minerals can be removed from the large pores. Experimental investigations using Hawkesbury sandstone samples were conducted by Rathnaweera et al. [78] to determine possible geochemical and mineralogical alterations upon CO_2_ injection. Using the inductively coupled plasma mass spectroscopy (ICP-MS), inductively coupled plasma atomic emission spectroscopy (ICP-AES), and SEM, the authors observed that over long-time intervals, the pore structure was altered, resulting in enhanced permeability. Christopoulou et al. [79] saturated three groups of sandstone from the Pentalofos formation in Greece with CO_2_ and brine, and estimated the mineralization behaviors by measuring the uniaxial compressive strength (UCS), bending stiffness, and Poisson's ratio to determine the optimal formation for the CO_2_ storage pilot testing. The authors investigated potential geological storage reservoirs in Greece in terms of mineralogical and mechanical properties of the sandstone specimens. The authors also proposed a petrographic index which quantifies the storage capacity of sandstones as a potential CCS candidate. Yasuhara et al. [80] artificially enhanced calcite precipitation in Berea sandstones by co-injecting urea, urease and CaCl_2_. It was found that in a 10 % substitution of the void space by the carbonate precipitation increased both the dynamic and static elastic moduli by twofold and 20 %, respectively. A 9 % decrease in permeability due to calcite precipitation. Fuchs et al. [70] analyzed the geomechanical property changes by injecting CO_2_- and N_2_-saturated brine into Mt. Simon sandstone specimens. As a result, the CT scanned images illustrated well-developed microfractures along the bedding planes and increased porosity, which indicate the rock was more affected by the CO_2_-saturated brine injection. The fracture toughness change suggests that the target reservoir might geomechanically weakened by CO_2_ injection. Huang et al. [81] injected CO_2_ into brine saturated Zunyi sandstone in Guizhou Province in China to investigate effects of brine salinity and CO_2_ phase on the UCS, Brazilian tensile strength (BTS), and fracture toughness. It was found that the 10.0–31.3 % of UCS, 30.5 % of BTS, and 27.2–50.0 % of fracture toughness were reduced by the scCO_2_ injections.

**Table 4:** Experimental studies focusing on the carbon mineralization in sandstone.

Author	Method	Lithology	Aqueous Matrix	Temperature (°C)	Pressure (MPa)	CO_2_ Phase	Time	Carbonate Precipitation	Remark
Wigand et al. (2008)	Core	Bunter Sandstone	1 M NaCl solution	60	15	scCO_2_	62.4 days (1496.9 h)	–	Injection of scCO_2_ in deep saline aquifers may show limited reactivity
Hangx et al. (2013)	Autoclave	Captain Sandstone, Goldeneye Field, Moray Firth, UK North Sea	Synthetic brine of goldeneye field	20 or 60	3.7–14	CO_2_(aq)	49 days (7 weeks)	–	Total dissolution of calcite did not affect rock mechanical properties
Marbler et al. (2013)	Autoclave	North German Basin	2 M NaCl	60 or 70	9.0–24.0	scCO_2_	14–28 days (2–4 weeks)	Calcite	Alterations occurred in both the carbonate and silicate cements of the sandstones.
Delle Piane and Sarout (2015)	Core	Berea sandstone	0.17 M NaCl	50	8	scCO_2_	–	–	Virtually unaffected mechanical properties and elastic moduli
Rathnaweera et al. (2016)	Core	Hawkesbury sandstone	3.4 M NaCl	40	2-6 (Injection pressure)	scCO_2_	548 days (1.5 years)	Halite	Pore structure alteration resulted in enhanced permeability and an increase in the effective stress coefficient
Al-Yaseri et al. (2017)	Core	Berea and Bandera Gray sandstones	0.85 M NaCl + 0.13 M KCl	49.85	10	scCO_2_	7 days	Calcite	Dissolution and precipitation in Berea and Bandera Gray sandstones resulted in minimal permeability changes
Yasuhara et al. (2017)	Core	Berea Sandstones	Co-injection of urea, urease and CaCl_2_	60	0.3 (Injection pressure)	CO_2_(g)	1 day (24 h)	Calcite	Carbonate precipitation substituted 10 % of the void space leading to an increase in both dynamic and static elastic moduli
Fuchs et al. (2019)	Core	Mt. Simon sandstone	Synthetic brine of Mt Simon formation pore water	50	22 (Injection pressure)	scCO_2_	28 or 56 days (4 or 8 weeks)	Calcite	Microfracturing which increase porosity were observed
Yu et al. (2019)	Autoclave & Simulation	Cretaceous Bashijiqike Formation (K1bs) of the Kuqa Depression in the Tarim Basin, China	Synthetic brine of K1bs Conditions	150	48.45	CO_2_(aq)	4,7,10,13,16 days	Kaolinite, Quartz	No significant change in core porosity after CO_2_ injection under high pressure (60 MPa confining pressure) and temperature (150 °C)
Huang et al. (2020)	Core	Zunyi sandstone in Guizhou Province, China	0, 1.7, 3.4, 5.1 M NaCl	32	10	scCO_2_	1 day (24 h)	–	UCS, BTS, and fracture toughness decreased for brine scCO_2_ injected sandstones
Pearce et al. (2021)	Core	Jurassic Precipice Sandstone in the Surat Basin, Australia	Deoxygenated synthetic produced water	50	0–10	scCO_2_	64.0 (1537 h)	Calcite, Dolomite	Observed a porosity change of 11.1–11.4 % after the reaction
Tang et al. (2021)	Core	Yinggehai Basin, DF gas fields in China	6 types of different brine	80	8	CO_2_(aq)	3 days (72 h)	Plagioclase	CO_2_-brine-rock interactions have a negative impact on CO_2_ injectivity and storage

On the other hand, there are studies suggesting that carbon mineralization in sandstone does not have a considerable impact during CCS. Wigand et al. [82] demonstrated the geochemical effects of CO_2_ in sandstones on in-situ reservoir pressures and temperatures over a total time span of 1496.9 h. The results suggested that the injection of scCO_2_ in deep saline aquifers may show limited reactivity with reservoir rocks and that the capillary and dissolution trap mechanisms are more dominating by dissolution of the injected scCO_2_. Yu et al. [83] injected CO_2_ dissolved brine into sandstone specimens from the Tarim Basin in China, and found that the CO_2_-brine-sandstone reaction only dissolved carbonates. It was concluded that there was no significant change in the rock porosity by the CO_2_ injection under high pressure (60 MPa confining pressure) and temperature (150 °C). Hangx et al. [73] performed CO_2_ injection into rock specimens extracted from the Goldeneye Field, Moray Firth, UK North Sea, and concluded that dissolved calcite did not change rock mechanical properties. Al-Yaseri et al. [75] reported that dissolution and precipitation in Berea and Bandera Gray sandstones induced permeability changes. Permeability of the Berea sandstone specimens was increased by 10∼37 %, while the Bandera Gray sandstone specimens indicate 3.1–18.8 % of decrease in permeability. Delle Piane and Sarout [84] compared the mechanical and elastic properties of dry, water- and scCO_2_-saturated Berea sandstone. The authors conducted triaxial experiments coupled with ultrasonic measurements and integrated the results with XRD and CT scanned images. The results show that virtually unaffected mechanical properties and elastic moduli at 50 °C and 18 MPa. Pearce et al. [35] performed injection of CO_2_ and water into specimens of the Jurassic Precipice sandstone in the Surat Basin, Australia, and observed carbonate precipitation by micro-CT, QEMSCAN, and SEM. It was concluded that the quartz-rich low-reactivity sandstones are mainly unaffected by mineralization in the 64.0 days (1537 h) with 0.3 % of the porosity change. However, it was found that the co-injection of CO_2_ with the produced water can accelerate the mineral trap, as the water can provide cations such as Ca^2+^, Mg^2+^, or Fe^2+^.

### 4.2 Numerical studies

Numerical studies conducted to identify detailed mechanisms of the carbon mineralization in sandstone are summarized in Table 5. Changes in composition induced by the mineralization have been observed in a few studies by computational approaches. Zhang and DePaolo [33] used mineralogy and measurements from the reactive surface area of the Nagaoka reservoir rock using the software package TOUGHREACT. It was concluded that the mineralization rate depends on both the abundance and release rate of the cations from the silicate minerals by dissolution accelerated by the low pH pore fluid. Using TOUGHREACT, Yu et al. [83] numerically modeled the Cretaceous Bashijiqike Formation in the Tarim Basin, China and observed that minor kaolinite and quartz precipitation. However, the experimental approach did not detect any evidence of the precipitation. Choi et al. [63] used TOUGHREACT to estimate the mineral trap rate in the Pohang Basin and concluded that iron-containing carbonate minerals, especially chlorite, significantly influenced the precipitation of siderite and ultimately the mineral trap capacity. Zerai et al. [85] performed computational simulations using Geochemist's Workbench to identify the mineralization behavior on the Rose Run carbonate, sandstone, and mixed mineral assemblages with various brine compositions. It was found that the CO_2_-brine-rock reaction induces dissolution of K-feldspar, annite, albite and kaolinite, and the precipitation of siderite, dawsonite and strontianite (SrCO_3_). The authors concluded that it is important to consider the dissolution and precipitation behavior of siderite and dawsonite to identify the effect of the mineral trap in the Rose Run Sandstone. Xu et al. (2004) incorporated TOUGHREACT to analyze the mineralization behavior for three aquifer mineral compositions. There was a 0.6 % decrease in the porosity after the CO_2_ injection. The results show that the carbonate accumulation reduces both the porosity and permeability of the aquifers. Sundal and Hellevang (2019) [86] adopted a computational approach to identify the mineralization behavior in the Johansen Formation, Norway. The results of the numerical simulation indicate that Na-plagioclase and Fe-chlorite are the main cation donors in the target formation. The authors concluded that the reactive surface area depends on the porosity, permeability, grain size and shape, sedimentary facies, and diagenetic imprint. Using CrunchFlow, Zhang et al. (2015) [87] discovered a permeability drop from 1.60 to 0.80 md in their experiment while the simulations indicated a drop from 1.60 to 1.02 md for the Mount Simon sandstone specimens. The authors concluded that the decrease in the permeability of was mainly caused by the SiO_2_ and kaolinite precipitated in the pore space as quartz and feldspar dissolved by injecting CO_2_ saturated brine. Xu et al. (2007) [88] constructed a numerical model to simulate co-injection of CO_2_ with H_2_S and CO_2_ with SO_2_ into a formation at 2 km depth in the Gulf Coast. At a reservoir temperature of 75 °C, it was found that the porosity increased from 0.30 to 0.43 after 100-year injection of the CO_2_–SO_2_ mixture, while there was no porosity change when the CO_2_ and H_2_S were co-injected. However, the CO_2_ and CO_2_–SO_2_ co-injection reduces the porosity from 0.3 to 0.28 at the CO_2_ mineral-trap zone and the acidification front, respectively. In addition, the porosities were decreased to 0.28 and 0.23 with the CO_2_–SO_2_ injection, respectively. Consequently, the precipitation of secondary carbonates and secondary sulfates altered both the rock porosity and permeability, which needs to be considered when CCS procedure is designed.

**Table 5:** Numerical studies of the carbon mineralization in sandstone.

Author	Method	Lithology	Aqueous Matrix	Temperature (°C)	Pressure (MPa)	CO_2_ Phase	Time	Carbonate Precipitation	Remark
Xu et al. (2004)	Numerical modelling	Glauconitic sandstone, Alberta Sedi-mentary Basin, United States Gulf Coast, and dunite	1 M NaCl	54 and 80	26	scCO_2_	1000 years	Ilite, Dawsonite, Ankerite, Magnesite	The accumulation of carbonates results in a decrease in porosity and affects the permeability and fluid flow
Zerai et al. (2006)	Numerical modelling	The Rose Run carbonate, sandstone, Ohio	4 different brine compositions of Ohio	35, 54, 75	20–26	scCO_2_	7000 years	Dawsonite, Siderite, Strontianite	Dissolution of albite, K-feldspar, and glauconite and the precipitation of siderite and dawsonite was observed
Xu et al. (2007)	Numerical modelling	Gulf Coast of the United States	0.975 M NaCl	75	10	scCO_2_	1000 years	Alunite	The porosity of the CO_2_ mineral trapping zone and the acidification front decreased to 0.28 and 0.23, respectively
Zhang et al. (2015)	Numerical modelling	Mount Simon sandstone	synthetic brine of Vermillion County, IN	85	42	scCO_2_	100 years	Illite, Muscovite	SiO_2_ and kaolinite formed in the pore space by the dissolution of quartz and feldspar
Choi et al. (2017)	Numerical modelling	The Pohang Basin	synthetic brine of Pohang Basin	29–43	5.2–8.8	scCO_2_	1000 years	Ilite, Kaolinite, Quartz, Smectite,	Iron-containing carbonate minerals significantly influence mineral trapping.
Zhang and DePaolo (2017)	Numerical modelling	Nagaoka reservoir rock	1 M NaCl	75	20	scCO_2_	1000 years	–	Rate of mineralization depends on both the abundance and rate at which cations are released from silicate mineral
Sundal and Hellevang (2019)	Numerical modelling	Johansen Formation, Norway, North Sea	synthetic brine of Johansen Formation	96	30	scCO_2_	10,000 years	Siderite, Magnesite	Reactive surface area depends on the grain size and shape, porosity, and permeability

## 5 The carbon mineralization in other rock types

### 5.1 Carbonate

Carbonate formations are also favorable candidates for geological CO_2_ storage [89]. It can be characterized by its high heterogeneity with poorly connected pore structures and complex fluid properties [90]. According to studies focusing on the CO_2_-EOR in carbonate formations, the CO_2_-water-rock interaction can occur actively upon the CO_2_ injection, because it mainly consists of dolomite, calcite and anhydrite (CaSO_4_) [91].

In addition, heterogeneous mineral dissolution and precipitation by the interaction may change pore structure, porosity and permeability of the rock, and formation fluid composition [92]. Because of the high heterogeneity, the interaction between CO_2_ and carbonate rock is still unclear. Moreover, alterations in geomechanical properties need to be considered during the carbon mineralization process in carbonate reservoirs [90].

Wang et al. [93] conducted laboratory experiments to evaluate the reactivity of dolomite in Southwest Wyoming by injecting water-saturated scCO_2_ for 96 and 164 h at temperature and pressure condition of 55, 110 and 220 °C, and 25 MPa, respectively. It was found that dolomite does not react with anhydrous supercritical carbon dioxide. Dolomite dissolves and carbonate minerals precipitate by reaction with water saturated scCO_2_ at 220 °C. Smith et al. [94] experimentally quantify the relationship between fluid flow and heterogeneity, and reaction specific to CO_2_ storage at Weyburn-Midale project in Canada. Experiment conditions are 60 °C and 24.8 MPa and brine composition were chosen to reservoir condition of Weyburn-Midale. Vuggy and Marly samples from the reservoir were used and X-ray computed microtomography (XCMT) and SEM revealed the complex mineral and pore structural within these samples. The impact of heterogeneity on pore space of carbonate rocks has a negative influence on CO_2_ pathway development. It made amplify the variability in fluid velocity and eventually unstable dissolution rate. Luquot et al. [95] performed CO_2_ injection experiments at 100 °C and 12 MPa using four limestone specimens extracted from the Mondeville formation in Paris basin in France. These experiments mimic mass transfer occurring near the injection well and far from injection well by ranging pCO_2_ from 0.7 to 10 MPa. The results indicate that non-uniform dissolution is dominating near the injection well (maximum pCO_2_), while uniform dissolution is obtained when pCO_2_ is 2.5 MPa. In addition, when pCO_2_ is 0.7 MPa that means far from the injection well, porosity decreased by the precipitation of Mg-rich calcite. Han al et [96]. visually investigated rock property alterations by CO_2_-water-rock interactions. CO_2_ was injected into an Edwards white limestone specimen from the Edwards plateau, Texas, and an Indiana limestone from Bedford, Indiana. The temperature and pressure conditions are maintained as 40 °C and 9.7 MPa, and the X-ray CT and SEM images were obtained before and after the injection experiments. In the case of the homogeneous Edwards white limestone, mineral dissolution facilitates the enlargement of pore sizes. For this reason, fluid through flow becomes more favorable due to the improvement of permeability (1.6–3.6 md). In the case of the heterogeneous Indiana limestone, both dissolution and precipitation happened and damage to the main flow channels through a reduction of permeability (74.5–24.1 md).

### 5.2 Shale

CO_2_ injection into shale has been studied primarily focusing on the enhanced hydrocarbon recovery and inducing artificial fractures [[97], [98], [99], [100]]. In addition, studies proposed that shale formations are also appropriate candidates for geological CO_2_ storage due to its high CO_2_ adsorption capacity and wide distribution [101]. It was found that the carbon mineralization in shale alters the chemical composition of the formation fluid and the pore structure of the layer [[102], [103], [104], [105]]. In addition, the changes result in the possible rock deformation and the weakened mechanical properties [102]. Therefore, not only may it alter the CO_2_ storage capacity, but it will also impact the storage stability of the target formation.

Lyu et al. [102] experimentally determined the CO_2_-water-shale interactions by gas phase CO_2_ and scCO_2_ injection into the Longmaxi shale specimens for 10, 20 and 30 days. The authors analyzed changes in the microstructure and the mechanical strength of the specimen. The results show that microstructure and composition were altered by dissolution of minerals, such as K-feldspar. In addition, it was found that the UCS, Youngs modulus and brittleness index were weakened with extended saturation time. When the specimens were saturated with the gas phase CO_2_-water, the UCS and the Young's modulus were respectively reduced 31.28 %–56.43 % and 27.39 %–54.21 % as the saturation time extended from 10 to 30 days. In addition, those were respectively decreased as much as 33.66 %–66.05 % and 30.27 %–56.32 % for the scCO_2_-water saturated specimens. It was found that the brittleness index was also reduced from 84.3 to 50.9 and 47.9 for the gas phase CO_2_-water saturated samples and scCO_2_-water saturated samples for the same saturation time. Ao et al. [103] investigated the rock property changes of the Longmaxi shale specimens by injecting gas phase CO_2_ for 5, 10, and 20 days. It was found that the specific surface, tensile strength, triaxial compressive strength and dynamic elastic moduli were reduced with the treatment time, while the porosity and average pore size were increased. When the specimens were saturated with scCO_2_ for 5 days, the specimens lost 22.7 % of the tensile strength, 15.3 % of the triaxial compressive strength, and 29.56 % of the elastic moduli at 6 MPa of confining pressure. Lahann et al. [104] experimentally investigated the influence of CO_2_ in the New Albany shale of the Illinois Basin and found that the Ca^2+^ and Mg^2+^ concentrations in filtrate fluids at the CO_2_ pore pressure of 24.13 MPa for 21 days. It was concluded the long-term effect of CO_2_ injection on New Albany shale is very complicated due to several mineral dissolution/precipitation. Feng et al. [105] performed the Brazilian splitting strength (BSS) test to identify the effect of the CO_2_ adsorption on the rock properties of the shale specimens extracted from the Sichuan Basin. By injecting scCO_2_ for 10, 30 and 60 days, it was found that BSS and splitting modulus decreases 11.3 %, 40.7 % and 45.7 % compared to the dry specimen. In addition, the XRD, XRF, and SEM images indicate microscopic alteration in the shale skeleton by scCO_2_-shale interaction. Kutchko et al. [106] investigated the interaction of CO_2_ in two Marcullus shale specimens by injecting scCO_2_ for 14 days at 10.3 MPa and 40 °C. It was concluded that the pore structure change by the interaction strongly depends on the total organic carbon, thermal maturity, and mineral composition. Yin et al. [100] measured the Energy dispersive X-ray spectroscopy and acoustic emission on Longmaxi shale specimens to investigate property change by the CO_2_-water-rock interaction. For the gas CO_2_ saturated specimens, the UCS and elastic moduli were decreased by 22.9 % and 23.1 %, respectively. On the other hand, 33.9 % of the UCS and 34.0 % of the elastic moduli were dropped by the scCO_2_ saturation. Consequently, it was concluded that the interaction strongly depends on the phase of the CO_2_.

## 6 Discussion

In this study, detailed mechanisms and behaviors of the carbon mineralization in basalt, sandstone, carbonate and shale were reviewed. During the geological CO_2_ storage, the carbon mineralization mainly depends on rock type, temperature, pressure, composition of formation fluid and injected fluid, competing reaction, nucleation, and mass transport limitation (Table 6). First, the divalent cation in the target formation enhances the mineralization process. Accordingly, the reactivity strongly depends on the rock type. In terms of temperature, precipitation occurs rapidly in siderite in low temperature, while the high temperature environment improves the precipitation in magnesite and ankerite. The pressure in the formation also affects the reactivity as precipitations of the Ca^2+^ and Mg^2+^ dominate at low- and high-pressure environment, respectively. If the formation has the low pH, the mineral dissolution is more dominating. However, the carbonate precipitation prevails in the high alkalinity environment. In addition, the competing reactivity between the carbon mineralization and silicate precipitation needs to be taken into account. If the silicate precipitation occurs, the rate of the carbon mineralization can be restricted by reduction of the pore space and divalent cations. Pre-existed carbonate mineral increases rate of the carbonate precipitation as it shortens the nucleation time. The location of the mineralization also affects the reactivity, as the mineral dissolution and precipitation would dominate in the advective flow and diffusive flow environments, respectively.

**Table 6:** The parameters effect on the carbon mineralization.

Parameter	Dominated by	Effects on the Carbon Mineralization
Rock type	Mineral composition, glass contents, grain size	Combined effect by divalent cation contained mineral and basaltic glass
Temperature	Storage condition	Low temperature: increases precipitation of Fe carbonate (siderite) High temperature: increases precipitation of Ca–Mg–Fe carbonate (magnesite, ankerite)
Pressure	Storage condition	Low pressure: increases precipitation of Ca carbonate (calcite, aragonite) High pressure: increases precipitation of Mg carbonate (magnesite)
Fluid composition	Formation water, injection fluid	Low pH: increases reactivity of mineral dissolution High alkalinity: increases reactivity of carbonate precipitation
Competing reaction	Temperature, Silicate and Clay mineral contents	Silicate precipitation: decreases pore space and divalent cations → decreases reactivity of carbonate precipitation
Nucleation	Mineral composition	Pre-existing carbonate mineral: decreases time spent on nucleation which is the rate determining step → increases reactivity of carbonate precipitation → decides carbonate mineral distribution
Mass transport limitations	Pore structure	Advective flow: decreases retention time and make low pH condition → increases reactivity of mineral dissolution Diffusive flow: increases retention time and make high pH condition → increases reactivity of carbonate precipitation

**Fig. 5:**
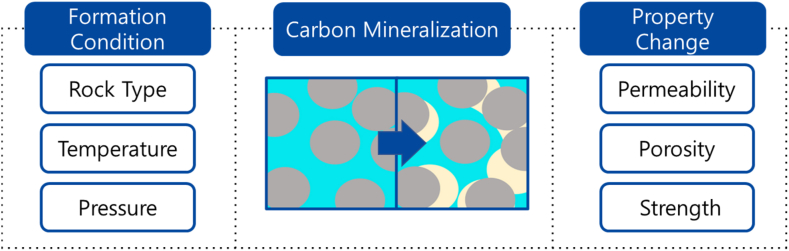
The carbon mineralization mechanism.

It was found that the process alters the reservoir properties including porosity and permeability of the target reservoir, and the rock mechanical properties including strength and elastic moduli. In addition, the properties of the formation fluid and the injected fluid can also be altered by the interaction between the rock-formation fluid-injected CO_2_ (Fig. 5). Since the changes can affect the important CCS parameters such as injectivity, storage capacity and stability, the carbon mineralization needs to be considered for the CCS design. Although it is widely accepted that the carbon mineralization does not occur in the early period in a low reactive rock such as sandstone, it should not be neglected as the long-term behavior of the injected CO_2_ needs to be considered. In addition, there are studies indicating that it needs to be considered when the rock contains sufficient divalent cations. Consequently, to investigate the long-term behavior of the carbon mineralization during CCS is required for safe and stable design.

Recently, basalt has been studied as a potential candidate for geological CO_2_ storage target, especially when the mineral trap is considered as the main trap mechanism. Since most studies were conducted before basalt was spotlighted as a candidate, those solely focus on the CO_2_-rock interaction. However, there are few studies addressing rock property change due to CO_2_ injection in basalt and its impact on the CCS design. On the other hand, studies dealing with sandstone are mainly focusing on rock properties change during CCS. This is because most studies are conducted for the enhanced hydrocarbon recovery design by injecting CO_2_ and water. As a result, future research of the mineralization in basalt should determine impact of the carbon mineralization on the CCS design. On the other hand, the detailed mechanisms of the carbon mineralization in sandstone should be identified in the future.

Injection schemes during CCS is another topic that needs to be studied. When CO_2_ is dissolved in water before injection, the carbon mineralization rate can be significantly accelerated. If the target formation does not have a confined structure, i.e., caprock, injecting the CO_2_ dissolved water can be a useful alternative. However, it requires a large volume of water with restricted CO_2_ volume, and additional cost for the process. For example, dissolving 1 ton of CO_2_ at a partial pressure of 25 bar and a temperature of 25 °C requires approximately 27 tons of water [19]. On the other hand, injecting scCO_2_ can be more economically efficient as the amount of the injected CO_2_ is significantly larger with a lower cost, although the carbon mineralization process is not considerate. Therefore, further research on the injection scheme for the optimal CCS design should be performed.

## 7 Conclusion

In this study, the carbon mineralization mechanism and behaviors in basalt, sandstone, carbonate, and shale were reviewed with relevant studies. The results of this study are as follows.

1) The carbon mineralization is defined by the CO_2_ reacted with divalent cations such as Ca^2+^, Mg^2+^ or Fe^2+^ to form stable carbonate minerals. Although most studies focus on rocks with high reactivity such as basalt, studies show that it can occur in other rock types including sandstone, carbonate, and shale. Since the mineral trap is a very important trapping mechanism in the long-term CO_2_ storage, the process should be considered regardless of rock type.
2) The carbon mineralization in basalt depends on temperature, pressure, fluid composition, silicate mineral precipitation, and nucleation. Related studies focus on the types of minerals formed, rates of mineralization, and geochemical reactions. For sandstone, the process strongly depends on the quartz content. This is because a higher quartz content can result in the limited availability of divalent ions. To identify detailed mechanism of the carbon mineralization is important for optimizing geological CO_2_ strategies. In addition, alteration of the rock properties by the carbon mineralization should be to ensure the effective and safe long-term CO_2_ storage.
3) The carbon mineralization mainly depends on rock type, temperature, fluid composition, and injected CO_2_ phase. Rock properties such as permeability, porosity, and rock strength can be altered by the carbon mineralization. Since changes of the properties are directly related to injectivity, storage capacity, and stability during the geological CO_2_ storage, the carbon mineralization mechanism should be considered for an optimal CCS design.

## Data availability statement

No data was used for the research described in the article.

## CRediT authorship contribution statement

**Kyuhyun Kim:** Writing – review & editing, Writing – original draft, Visualization, Investigation, Conceptualization. **Donghyun Kim:** Writing – review & editing, Writing – original draft. **Yoonsu Na:** Writing – review & editing, Writing – original draft. **Youngsoo Song:** Writing – review & editing, Writing – original draft. **Jihoon Wang:** Writing – review & editing, Writing – original draft, Conceptualization.

## Declaration of competing interest

The authors declare that they have no known competing financial interests or personal relationships that could have appeared to influence the work reported in this paper.
